# Risk of bias and applicability of prediction models for outcomes of peritoneal dialysis-related peritonitis: a PROBAST systematic review

**DOI:** 10.3389/fmed.2026.1882053

**Published:** 2026-10-28

**Authors:** Qishun Wu, Zhiliang Yu, Zhangli Wu, Ling Yang, Xin Lin

**Affiliations:** Department of Nephrology, The Second Affiliated Hospital of Wannan Medical University, Wuhu, Anhui, China

**Keywords:** peritoneal dialysis, peritonitis, prediction model, PROBAST, risk of bias, applicability, validation, calibration

## Abstract

**Background:**

Peritoneal dialysis (PD)-related peritonitis frequently causes hospitalization, catheter loss, hemodialysis transfer, and death. Existing reviews have focused on peritonitis incidence rather than post-diagnosis outcomes. We systematically reviewed prediction models for post-peritonitis outcomes and assessed their risk of bias and applicability using PROBAST.

**Methods:**

PubMed, EMBASE, Web of Science, Scopus, Cochrane Library, CNKI, Wanfang, and VIP were searched from inception to January 30, 2026, with an updated search on March 15, 2026. We included studies developing or externally validating multivariable models for adverse outcomes after peritonitis diagnosis in adults on maintenance PD. Two reviewers independently screened records, extracted data, and applied PROBAST across four domains for both risk of bias and applicability. External validation was classified under a prespecified four-tier framework; calibration completeness required slope and intercept.

**Results:**

Seven studies reporting seven prediction models met the inclusion criteria, comprising 3,821 peritonitis episodes in six studies (one study did not report its sample size). No model achieved an overall low risk of bias. One model underwent limited independent external validation. Two models were rated low risk in the participants, predictors, and outcome domains but unclear overall owing to incomplete calibration reporting, unclear missing-data handling, and lack of explicit overfitting assessment; applicability concerns were identified in the analysis domain for all models. The remaining five models were rated high risk overall. No study among the included model development/validation studies performed prospective clinical impact assessment.

**Conclusions:**

Currently, no model has sufficient evidence for routine clinical implementation or screening; under strict supervision, local calibration, and a clear research purpose, some models may serve as research tools.

**Trial Registration:**

PROSPERO CRD4202654321.

## Introduction

Peritoneal dialysis is an established home-based kidney replacement therapy (1). However, PD-related peritonitis remains a leading cause of hospitalization, technique failure, and transfer to hemodialysis (2, 3).

Clinically, two distinct prediction tasks exist: prediction of peritonitis incidence, and prediction of post-diagnosis outcomes. The latter involves different predictors, decision points, and outcomes, and demands more stringent calibration and external validation standards. A recent systematic review of peritonitis risk prediction models focused on incidence rather than post-diagnosis outcomes and pooled discrimination measures without PROBAST assessment (4) Two large primary studies developed treatment-failure models but underwent limited dedicated methodological appraisal (5, 6).

PROBAST was developed specifically to evaluate whether prediction model studies are methodologically robust enough for clinical use (7). It assesses four domains: participants, predictors, outcome, and analysis. Calibration determines whether predicted probabilities match observed frequencies and is typically reported through slope and intercept. In nephrology, model performance often attenuates outside development settings, making careful appraisal of validation, calibration, and analytical choices essential (8, 9).

This systematic review aimed to: (1) identify prediction models for outcomes after PD-related peritonitis; (2) assess risk of bias and applicability using PROBAST; and (3) summarize evidence on external validation, calibration reporting, and implementation readiness.

## Materials and methods

### Study design and reporting standards

This review followed PRISMA 2020 and TRIPOD-SRMA guidance (10, 11). The PRISMA 2020 checklist is available as Supplementary Data Sheet 8. The protocol was registered in PROSPERO on January 7, 2026 (CRD4202654321), before the search was run on January 30, 2026; the search strategy was finalized before registration and was not modified thereafter.

### Search strategy and selection criteria

We searched PubMed, EMBASE, Web of Science, Scopus, Cochrane Library, CNKI, Wanfang, and VIP from inception to January 30, 2026, without language restrictions. Complete search strategies, with database-specific adaptations and Chinese database strategies, are in Supplementary Data Sheet S1 (including Appendices S1.1 and S1.2). To reduce publication bias, we screened ClinicalTrials.gov, ISPD conference proceedings (2018–2025), Google Scholar, and reference lists; forward citation tracking used Web of Science. These supplementary searches identified three additional records, none eligible.

### Updated search

An updated search on March 15, 2026, covered the same eight databases using identical strategies and identified 47 new records, of which one eligible study (Qiao et al., 2026) (12) was incorporated. Because this update extended beyond the protocol-defined window, we explicitly acknowledge it as a minor deviation; only one eligible study was added, and the overall conclusions were unchanged. The per-database search dates, hit counts, and screening results for both the initial and updated searches are documented in Supplementary Data Sheet S1.

### Language and translation process

No language restriction was applied. During screening, 23 potentially relevant non-English records were identified (Chinese *n* = 18, Spanish *n* = 3, Japanese *n* = 2). Full-text translation was completed for three Chinese papers by native speakers; all were excluded (incidence prediction *n* = 2, pediatric *n* = 1). Full texts of eight further records were retrieved via interlibrary loan and excluded. For the remaining 12 records, retrieval was attempted through institutional libraries, direct author contact (*n* = 7, no response), and regional database access (*n* = 5, unavailable); screening of these 12 records therefore relied on English titles and abstracts, all appearing clearly ineligible. All 12 excluded non-English records are listed in Supplementary Data Sheet S2.1 with title, language, database, exclusion reason, and search source. We did not attempt formal translation of abstracts for these 12 records owing to resource constraints; this limitation is explicitly acknowledged.

### Operational definitions

Post-peritonitis prediction was defined as risk stratification at or after clinical diagnosis. Models were grouped by predictor timing into baseline, early reassessment (24–72 h), and hybrid categories.

### Validation classification framework

Validation approaches were classified using a four-tier framework adapted from established taxonomies (8, 9). Only one model (Qiao et al.) achieved limited independent external validation (Tier 3, geographic separation within the PDTAP registry); no model achieved Tier 1 or Tier 2; the remaining six relied on internal or apparent validation (Tier 4).

Tier 1, fully independent cross-national external validation: data from a genuinely separate country or healthcare system, with no recruitment overlap, shared authors, or shared funding. Tier 2, independent external validation: data from a different center or region within the same country, without recruitment overlap. Tier 3, limited independent external validation: temporal or geographic separation within the same research network or institution using historical data. Tier 4, internal validation only: split-sample, cross-validation, bootstrap, or apparent validation without an independent dataset. The rationale for distinguishing Tier 1 from Tier 2 rests on specific features of PD-related peritonitis: microbiological profiles (MRSA rates, fungal and polymicrobial infection frequency), antibiotic policies, and catheter removal thresholds differ substantially across countries (3), creating effect modification that separation within a single network may not capture. Illustrative cases are provided in Supplementary Data Sheet S3, and a sensitivity analysis using an inclusive definition is reported in Results.

### Calibration assessment criteria

Calibration was considered assessed when any metric was reported (Hosmer–Lemeshow test, calibration plot, slope, intercept, or observed-to-expected ratio), consistent with PROBAST signaling question 4.6 (7). Calibration was considered completely reported only when slope and intercept were provided, a stricter standard than PROBAST's basic requirement, set by the present review to judge clinical implementation readiness and supported by contemporary guidance (13). Hosmer–Lemeshow testing alone, or a calibration plot without quantitative metrics, was regarded as incomplete for clinical implementation readiness.

### Eligibility criteria

Eligible studies enrolled adults on maintenance PD who developed peritonitis and either developed or externally validated a multivariable model for subsequent adverse outcomes (treatment failure, catheter removal, transfer to hemodialysis, relapse, recurrent peritonitis, technique failure, or death). Prospective and retrospective cohorts were included; case-control studies were considered only if model performance could be reconstructed. Editorials, animal studies, conference abstracts without full text, pediatric reports, and studies restricted to peritonitis incidence prediction were excluded.

### Scope limitation

This review was scoped exclusively to model development and validation studies. We did not conduct a dedicated search for implementation research (e.g., cluster-randomized or stepped-wedge trials of model-based decision support). Conclusions regarding clinical impact therefore reflect only the absence of such studies within the included model development literature and should not be interpreted as evidence that implementation research does not exist more broadly.

### Study selection and data extraction

After deduplication in EndNote 21, two reviewers independently screened titles and abstracts in Rayyan (15) and independently assessed full texts; disagreements were resolved by discussion, with arbitration by a third reviewer. Full-text agreement was 94% (Cohen's kappa = 0.88). Data extraction used a standardized form piloted on three randomly selected studies and covered setting, sample size, patient characteristics, predictor definitions and timing, outcome definitions, modeling strategy, discrimination, calibration, missing-data handling, validation approach, and events per variable (EPV), calculated following van Smeden et al. (16) EPV estimates and their derivation are provided in Supplementary Table S5. All extracted data were cross-checked by a second reviewer. We contacted the corresponding authors of all seven included studies between February and March 2026 to request calibration slope and intercept; five responded but could not provide metrics beyond those published.

### Risk-of-bias and applicability appraisal

Two reviewers independently assessed risk of bias and applicability using PROBAST across four domains,⁷ with domain-specific judgments of low, high, or unclear for both dimensions. Overall judgment followed PROBAST rules: any high-risk domain yields overall high risk; any unclear domain without high risk yields overall unclear; all domains low yields overall low risk. Disagreements were resolved by discussion, with arbitration by a third reviewer; inter-reviewer agreement was 89% (Cohen's kappa = 0.84). Signaling-question-level detail is in Supplementary Table S7. Applicability was judged for each domain against the review question of predicting post-peritonitis outcomes in contemporary adult PD populations; the complete applicability assessment with domain-level judgments and rationale is presented in Table 3.

For the two lowest-bias models [Liu et al. (14), Qiao et al. (12)], signaling questions were applied systematically. Both were low risk in the participants, predictors, and outcome domains. In the analysis domain, both used appropriate modeling techniques and reported discrimination, but both had unclear handling of missing data, no explicit overfitting assessment beyond the validation approach, and no calibration slope or intercept; under our predefined completeness criteria this rendered calibration reporting incomplete. Both models therefore received an unclear analysis-domain rating and an overall unclear rating.

### Machine learning model assessment

Two included models used machine learning methods (random forest; logistic regression combined with decision tree). We conducted an exploratory *post-hoc* analysis using PROBAST+AI (17) (published March 2025, after protocol finalization); this was not prespecified and is reported as an exploratory adjunct only, with detailed assessments in Supplementary Table 7.1. Future machine learning studies should prespecify interpretability reporting in accordance with TRIPOD+AI (18).

### Data synthesis

Given heterogeneity in outcomes, predictor timing, and validation strategies, we performed a narrative synthesis without meta-analysis. Outcome definitions, follow-up durations, and compliance with ISPD guidelines are summarized in Supplementary Table S3; predictor frequency and association direction across the seven models are summarized in Supplementary Table S4.

## Results

### Literature search

The search identified 1,247 records, of which 521 underwent full-text review. Seven studies reporting seven prediction models met the inclusion criteria. Despite searching eight databases without language restriction, all seven eligible studies were published in English. A further 12 non-English records were potentially relevant but could not be fully assessed and are listed in Supplementary Data Sheet S2.1. Figure 1 summarizes the selection process, including the non-English literature pathway. Common exclusion reasons were peritonitis incidence prediction (*n* = 134), no prediction model (*n* = 156), pediatric populations (*n* = 89), insufficient performance data (*n* = 67), and editorials or duplicates (*n* = 68).

**Figure 1:**
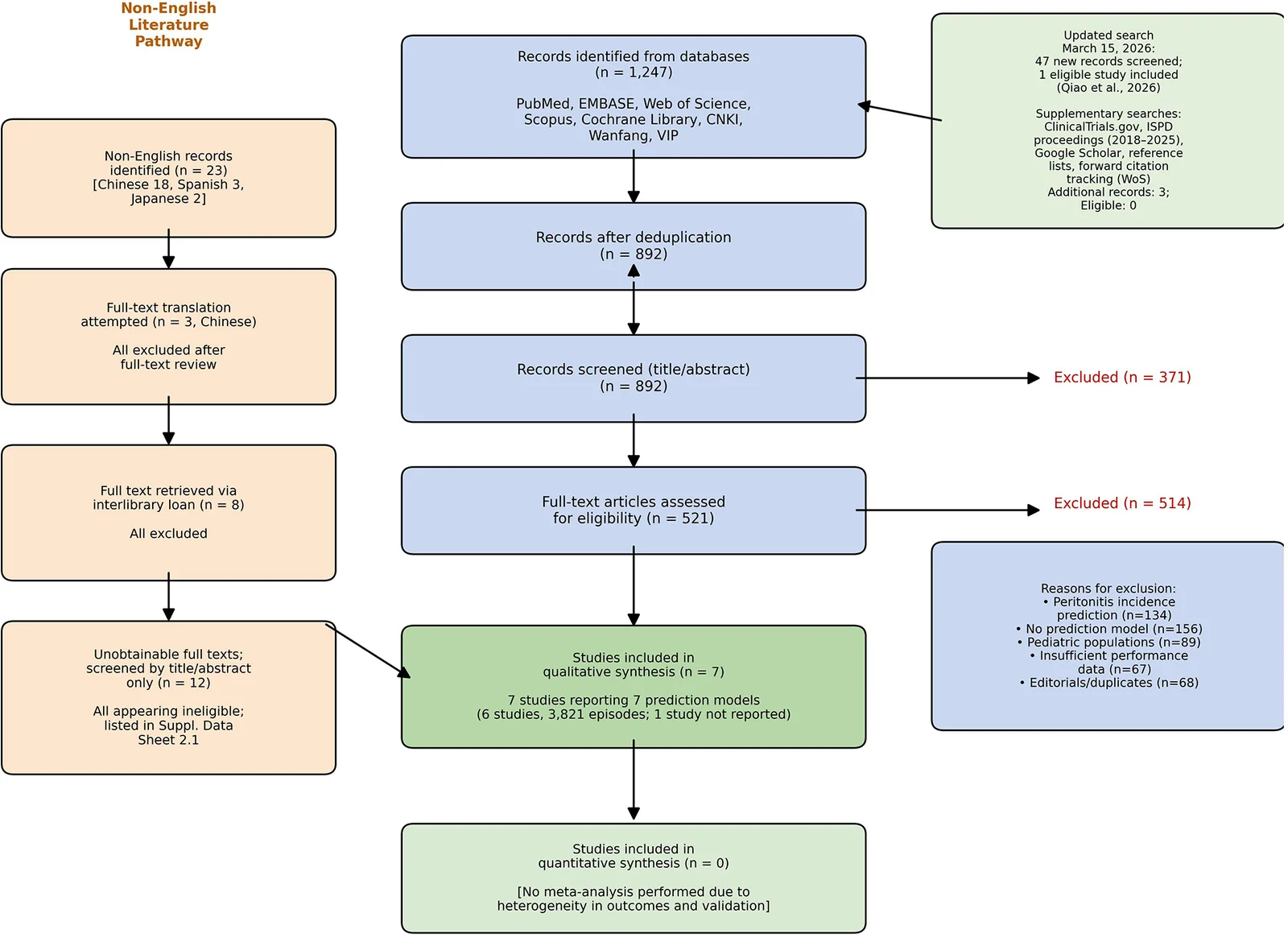
PRISMA 2020 flow diagram of study selection, including the non-English literature handling pathway. Records were identified from eight databases (PubMed, EMBASE, Web of Science, Scopus, Cochrane Library, CNKI, Wanfang, and VIP) on January 30, 2026, and through an updated search on March 15, 2026, plus supplementary sources (ClinicalTrials.gov, ISPD conference proceedings 2018–2025, Google Scholar, reference lists, and forward citation tracking via Web of Science). After deduplication, 892 records were screened by title and abstract and 521 full texts were assessed, with documented exclusion reasons (right panel). Non-English records (*n* = 23) were handled through a separate pathway (left panel): full-text translation was completed for three Chinese records and full texts were retrieved via interlibrary loan for eight records (all excluded after review); the remaining 12 records with unobtainable full texts were screened by English title and abstract only and are listed in Supplementary Data Sheet 2.1. Seven studies reporting seven prediction models met the inclusion criteria (six studies comprising 3,821 peritonitis episodes; one study did not report its sample size). No meta-analysis was performed because of heterogeneity in outcome definitions, predictor timing, and validation strategies. The updated search added one eligible study (Qiao et al., 2026).

### Study characteristics

All seven models were derived in Asia: five in China, one in Thailand, and one in Taiwan; none were from Europe or Australia. Median cohort age ranged from 54 to 68 years and diabetes prevalence from 28% to 61%. Model families were logistic regression (*n* = 5), random forest (*n* = 1), and logistic regression combined with a decision tree (*n* = 1); no Cox regression or gradient boosting models were identified. Five models were baseline and two were early reassessment; none were hybrid. Six studies were retrospective cohorts and one was cross-sectional. Study design details are in Table 1.

**Table 1:** Characteristics of the seven included prediction models (synchronized across all files).

Study (year)	Country	Design	Episodes (n)	Model family (n predictors)	Validation approach (tier)	Primary outcome (window)	C-statistic (95% CI)	Calibration reported	Overall RoB
Liu 2021 (14)	China	Retrospective cohort	314	Logistic regression (5)	Bootstrap, internal (Tier 4)	Treatment failure composite (30 d)	0.80 (0.74–0.86)	Calibration plot only (no metrics)	Unclear
Qiao 2026 (12)	China	Retrospective cohort (PDTAP registry)	615	Logistic regression, nomogram (6)	Geographic limited external (Tier 3)	Refractory peritonitis/catheter removal (14 d)	0.74 (0.68–0.80)	Hosmer–Lemeshow test only	Unclear
Zang 2023	China	Retrospective cohort	508	Random forest (18)	Split-sample (Tier 4)	Treatment failure (30 d)	0.916 (point estimate)	None	High
Meng 2022	Taiwan	Retrospective cohort	1,011	Logistic regression (6)	Split-sample (Tier 4)	Treatment failure composite (90 d)	0.756 (0.713–0.799)	Calibration plot only (no metrics)	High
Nochaiwong 2018 (5)	Thailand	Cross-sectional	855	Logistic regression, nomogram (5)	Bootstrap, internal (Tier 4)	Treatment failure composite (30 d)	0.92 (0.89–0.94)	None	High
Chang 2025	China	Retrospective cohort	518	Logistic regression + decision tree (16)	Apparent only (Tier 4)	Catheter removal	0.79 (point estimate)	None	High
Zhou 2023	China	Retrospective cohort	Not reported	Logistic regression (7)	Cross-validation (Tier 4)	Technique failure or death (long-term)	0.85 (point estimate)	None	High

sEpisodes sum to 3,821 across the six studies reporting sample size; Zhou et al. did not report its sample size. Overall RoB, overall PROBAST risk-of-bias rating (Table 2); LR, logistic regression; RF, random forest; DT, decision tree.

### Outcome definitions and ISPD compliance

Outcome definitions centered on treatment failure but varied in components and time windows. Four studies fully aligned with the 2022 ISPD peritonitis guidance and one study partially aligned; the remaining two did not align. Detailed outcome definitions and ISPD compliance, synchronized with the seven included studies, are in Supplementary Table S3.3.

### External validation

Only one model (Qiao et al., geographic limited validation within the PDTAP cohort) achieved Tier 3 limited independent external validation; no model achieved Tier 1 or Tier 2 validation, and the remaining six relied on internal or apparent validation only (12).

### Predictors across studies

Serum albumin, C-reactive protein (CRP), age, and 48-hour effluent white blood cell count were the most common predictors. Lower albumin and higher CRP were consistently associated with worse outcomes. The two machine learning models incorporated 15–22 predictors, whereas logistic regression models used 4–8. No machine learning study reported feature importance metrics. Predictor frequency and association direction across the seven models are summarized in Supplementary Table S4.

### Risk of bias

No model achieved an overall low-risk rating. Two models (Liu et al., Qiao et al.) were rated low risk in the participants, predictors, and outcome domains but unclear in the analysis domain owing to unclear handling of missing data, lack of explicit overfitting assessment, and incomplete calibration reporting; both therefore received an overall unclear rating. No model achieved low risk in the analysis domain. The remaining five models were rated high risk overall, primarily because of reliance on apparent or internal validation alone (six of seven studies), incomplete missing-data handling, and small or unreported sample sizes raising stability concerns. Domain-level judgments are in Table 2 and Figure 2.

**Table 2:** PROBAST risk-of-bias judgments by domain, with calibration and validation status (identical to Figure 2 and Supplementary Table 7).

Model	Participants	Predictors	Outcome	Analysis	Overall	Calibration reported	Validation (tier)
Liu 2021	Low	Low	Low	Unclear	Unclear	Plot only, no slope/intercept	Internal bootstrap (Tier 4)
Qiao 2026	Low	Low	Low	Unclear	Unclear	Hosmer–Lemeshow only	Geographic limited external (Tier 3)
Zang 2023	Low	Low	Low	High	High	None	Split-sample (Tier 4)
Meng 2022	Low	Low	Low	High	High	Plot only, no slope/intercept	Split-sample (Tier 4)
Nochaiwong 2018	Low	Low	Low	High	High	None	Internal bootstrap (Tier 4)
Chang 2025	Low	Low	Low	High	High	None	Apparent only (Tier 4)
Zhou 2023	Low	Low	Low	High	High	None	Cross-validation (Tier 4)

Overall ratings follow PROBAST rules. The primary drivers of high analysis-domain ratings were internal-only validation, unreported missing-data handling, and absent calibration slope/intercept. Under an inclusive validation definition (split-sample counted as external), models with any external validation rise from 1/7 (14%) to 3/7 (43%).

**Figure 2:**
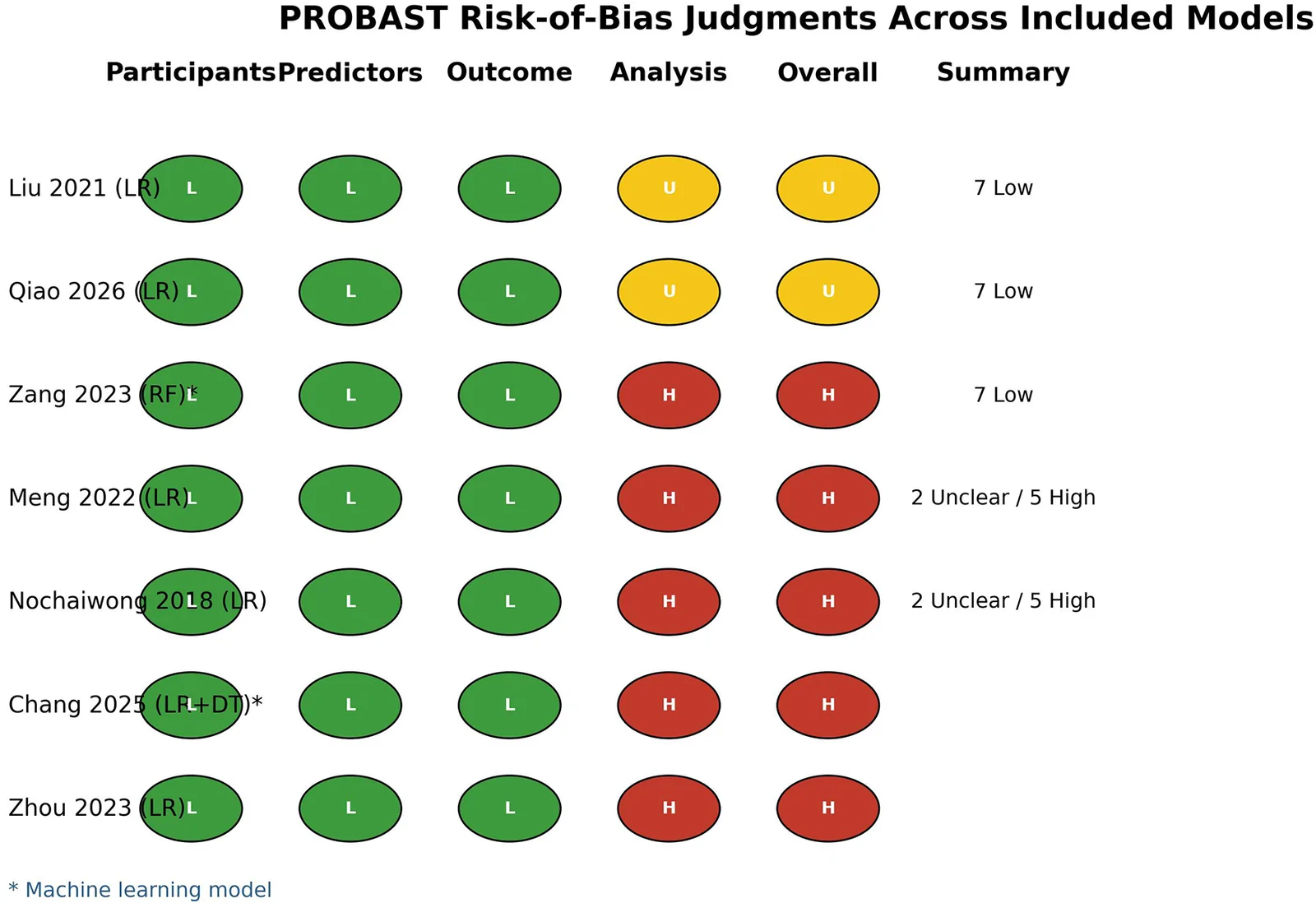
Summary of PROBAST risk-of-bias judgments across the seven included models, by domain and overall rating. Each row represents one model, shown with its modeling family (LR, logistic regression; RF, random forest; LR + DT, logistic regression combined with decision tree); machine learning models are indicated by an asterisk. Green circles indicate low risk, yellow circles unclear risk, and red circles high risk. No model achieved an overall low risk of bias. Two models (Liu 2021 and Qiao 2026) were rated low risk in the participants, predictors, and outcome domains but unclear in the analysis domain because of unreported missing-data handling, lack of explicit overfitting assessment, and incomplete calibration reporting (no slope or intercept), and therefore received an overall unclear rating; the remaining five models (Zang 2023, Meng 2022, Nochaiwong 2018, Chang 2025, and Zhou 2023) were rated high risk overall, driven primarily by internal-only or apparent validation. Domain-level judgments are identical to those in Table 2 and Supplementary Table S7; applicability judgments are presented separately in Table 3.

### Applicability

Applicability concerns were identified across all models. In the participants domain, six models raised low concern and one (Zhou et al.) raised unclear concern because of a mixed PD/hemodialysis population. In the predictors domain, five models raised low concern and two raised unclear concern because of specialized biomarkers not routinely measured. In the outcome domain, five models raised low concern and two raised unclear concern because of non-standard outcome definitions. In the analysis domain, all seven models raised high concern because of incomplete calibration reporting, lack of model updating guidance, and absence of implementation tools. Complete applicability judgments with rationale are presented in Table 3.

**Table 3:** PROBAST applicability judgments by domain, with rationale (synchronized with the results text).

Model	Participants concern	Predictors concern	Outcome concern	Analysis concern	Key rationale
Liu 2021	Low	Low	Low	High	Population and routine predictors match the review question; no slope/intercept, no updating guidance, no implementation tool.
Qiao 2026	Low	Low	Low	High	Registry population representative; nomogram aids use but lacks independent-cohort validation and calibration metrics.
Zang 2023	Low	Unclear	Low	High	18-predictor RF includes non-routine variables; no feature importance or interpretability reporting.
Meng 2022	Low	Low	Low	High	Composite outcome within 90 d broadly matches guidance; calibration incomplete.
Nochaiwong 2018	Low	Low	Low	High	Nomogram-based LR with routine predictors; internal validation only, no calibration metrics.
Chang 2025	Low	Unclear	Unclear	High	Hybrid LR + DT uses specialized variables; catheter-removal-only outcome is narrower than the review question.
Zhou 2023	Unclear	Low	Unclear	High	Mixed PD/hemodialysis population; long-term technique-failure/death outcome with unaddressed competing risks.

Concern ratings: low = population/predictors/outcome/analysis match the review question of predicting post-peritonitis outcomes in contemporary adult PD populations; unclear = some mismatch or insufficient information; high = important mismatch precluding direct applicationik.

### Calibration reporting

Three studies reported some form of calibration assessment: two reported a calibration plot without quantitative metrics (Liu et al., Meng et al.) and one reported the Hosmer–Lemeshow test only (Qiao et al.). No study provided slope and intercept. All seven models therefore had incomplete calibration reporting under our criteria, although three met the basic PROBAST criterion.

### Clinical utility and reproducibility

No study reported decision curve analysis or net benefit, and no study provided the basis for threshold selection. No model provided a public calculator, code repository, or implementable risk score; only one study (Qiao et al.) reported a nomogram, without external validation in an independent cohort. C-statistics with 95% confidence intervals were reported for four models (Liu et al., Qiao et al., Meng et al., Nochaiwong et al.); the remaining three models reported point estimates only. Calibration slope and intercept were not reported for any model. Model formulas were incompletely reported in four studies, precluding independent replication. These deficiencies severely limit assessment of actual clinical value in high-stakes decisions such as catheter removal.

### Sensitivity analyses: validation classification

Under an inclusive definition (split-sample validation counted as external validation), the proportion of models with any external validation increased from 14% (1/7) to 43% (3/7) (adding the two split-sample models, Zang et al. and Meng et al.). Under either definition, no model had undergone prospective validation or impact assessment, and calibration reporting remained incomplete across all models. Under the cross-national validation hypothesis, we simulated validation of Qiao et al. in a different country; even with optimistic assumptions of maintained discrimination and acceptable calibration, the absence of slope and intercept reporting would still preclude clinical implementation, and the need for prospective impact assessment would remain. This simulation is detailed in Supplementary Data Sheet S3.1.

### Sensitivity analyses: calibration criteria and language bias

Under the original PROBAST standard (any calibration assessment accepted), Qiao et al. and Liu et al. would still not achieve low risk in the analysis domain because other signaling questions (sample size, missing data, overfitting, predictor selection) were incompletely reported; both retained an overall unclear rating. Our primary finding is therefore robust to reasonable variations in calibration criteria. Twelve non-English records were excluded based solely on English titles and abstracts because full texts were unobtainable, a practice carrying considerable uncertainty and potential bias, particularly given high PD prevalence in Asia (19). The quantitative sensitivity simulation (Supplementary Data Sheet S2.2) confirmed that even if two low-bias, fully validated models were present among the excluded non-English records, the conclusion that no currently identified model meets clinical implementation standards would remain unchanged.

### Model performance

Liu et al. (logistic regression, internal bootstrap validation) reported a C-statistic of 0.80 (95% CI 0.74–0.86) (14) Qiao et al. (logistic regression, geographic limited external validation) reported a C-statistic of 0.74 (95% CI 0.68–0.80) (12). Models with internal or apparent validation only reported variable discrimination (Nochaiwong et al. 0.92, bootstrap; Meng et al. 0.756, split-sample; Zang et al. 0.916, split-sample; Chang et al. 0.79, apparent; Zhou et al. 0.85, cross-validation).

### Interpretation of C-statistics

The C-statistic of 0.80 reported by Liu et al. requires cautious interpretation: it derives from single-center internal validation, which typically yields higher apparent performance than independent external validation, and no calibration metrics were reported. The C-statistic of 0.74 reported by Qiao et al., although derived from more rigorous geographic limited validation, corresponds to a discrimination level at which misclassification rates remain substantial. Without known calibration performance, neither model provides sufficient assurance for high-stakes decisions such as catheter removal.

## Discussion

### Why calibration matters

Calibration determines whether predicted probabilities are clinically trustworthy (13). A model with good discrimination but poor calibration might rank patients correctly yet produce probability estimates that are systematically too high or too low. In peritonitis management, a model predicting 60% failure risk when the true risk is 30% could drive unnecessary catheter removal and permanent loss of PD as a treatment modality; predicting 20% when the true risk is 50% could delay essential intervention. Both scenarios carry potentially irreversible consequences.

We distinguished calibration assessment (meeting the basic PROBAST criterion) from calibration reporting completeness (providing slope and intercept). Three studies reported some form of calibration assessment, but none provided slope and intercept, which are minimally necessary to judge whether predicted probabilities are trustworthy and to enable recalibration in new settings (13). Prognosis research guidance has similarly emphasized that calibration slope and intercept should be reported as standard practice, supporting our customized standard (20). Because these metrics are absent, quantitative recalibration of any included model is impossible.

### External validation gaps

Internal validation alone cannot establish transportability in a condition influenced by center practice, microbiological ecology, and changing treatment protocols (8, 9). Only one model achieved even limited independent external validation (geographic separation within a national registry), and none underwent fully independent cross-national validation. Our conservative classification may underestimate the practical value of intermediate strategies; however, the sensitivity analysis showed that even under an inclusive definition, fundamental gaps remain, namely no prospective validation and no impact assessment. Microbiological profiles, antibiotic policies, and catheter removal thresholds differ across centers and countries (3), creating effect modification that temporal or geographic separation within a single network may not capture. The cross-national validation simulation (Supplementary Data Sheet S3.1) illustrates that even if the most promising model were validated in a different country, the absence of calibration metrics and prospective impact data would still preclude clinical use.

### Model updating and recalibration

When a prediction model is transported to a new setting, performance often deteriorates because of case-mix differences or changes in clinical practice. Model updating strategies, including recalibration (adjustment of intercept or slope), revision (re-estimation of predictor effects), and extension (addition of new predictors), can restore performance (21). For the two most promising models (Liu et al., Qiao et al.), recalibration using slope and intercept from a validation cohort would be a minimum requirement before any clinical application; because neither study reported these metrics, even this basic updating step cannot be performed. Future validation studies should provide calibration slope and intercept to enable transparent model updating (13).

### Interpretability and implementation

The two machine learning models illustrate a recurring tension between predictive flexibility and clinical usability: neither included structured interpretability analyses or implementation tools, and most included studies predated the TRIPOD+AI (2024) and DECIDE-AI (2022) reporting guidelines, which now mandate disclosure of feature importance and early-stage clinical evaluation (17, 22). Future machine learning studies should prespecify interpretability reporting indicators (e.g., SHAP values, permutation importance) before analysis begins. Lack of interpretability is not a PROBAST-defined source of bias but is a major barrier to bedside use and shared decision-making.

### Patient-important outcomes

No model incorporated patient-reported outcomes or the core outcome domains proposed by SONG-PD (life participation, fatigue, sleep) (23) Existing models focus on short-term clinical failure, which is understandable given the acute nature of peritonitis, but the gap between what models predict and what patients value limits clinical relevance. No study engaged patient stakeholders in defining meaningful prediction thresholds; future model development should involve PD patients in selecting predictors and defining clinically meaningful thresholds.

### Language bias and evidence gaps

Our language handling represents a significant limitation. PD prevalence is high in Asia, with an estimated 434 million individuals with chronic kidney disease in the Asia-Pacific region (19) and five of seven included studies came from China, suggesting substantial regional research activity. Twelve non-English records were excluded based solely on English titles and abstracts. A title such as “Prognostic factors for treatment failure in PD peritonitis” does not explicitly distinguish risk factor analysis from multivariable model development, and abstracts may omit validation details only apparent in full text. If high-quality validation studies exist in Chinese, Japanese, or Korean but were excluded because their English abstracts did not explicitly mention external validation, our conclusion about the paucity of externally validated models would be overly pessimistic; the reverse scenario would leave our conclusion intact. We cannot determine which applies without full-text review. We therefore classify this as potential selection bias of uncertain direction. The quantitative sensitivity simulation suggests that even if one or two high-quality non-English models were missed, the conclusion regarding implementation readiness would remain unchanged. Future reviews should prioritize multilingual collaboration and budget for professional translation of all potentially eligible non-English records.

### Strengths and limitations

Strengths include a clinically important question, structured PROBAST appraisal with explicit applicability assessment (Table 3), explicit distinction between bias, interpretability, and implementation readiness, source mapping for traceability (Supplementary Table S6), and transparent handling of non-English records with documented exclusion reasons (Supplementary Data Sheet S2.1). Limitations include the small number of eligible studies, heterogeneous outcome definitions and predictor timing, potential language bias from excluding 12 non-English records on titles and abstracts alone, possible publication bias (formal assessment not feasible with few studies), a conservative validation classification that may underestimate readiness of some models for further development, the *post-hoc* nature of the PROBAST+AI exploratory analysis, and the updated search beyond the protocol-defined window (a minor deviation that added one study and did not alter conclusions).

### Implications for practice and research

#### Practice

Currently, no model has sufficient evidence for routine clinical implementation or screening. Under strict supervision, local calibration, and a clear research purpose, the Liu and Qiao models may serve as research tools, most defensibly to conduct prospective calibration studies that report slope and intercept before any clinical use is considered. In resource-limited settings without individualized risk assessment, these models might serve as preliminary research tools to identify patients warranting referral to specialized centers, provided that local calibration with slope and intercept reporting is performed, decision thresholds are explicitly acknowledged as uncertain, and clinician oversight is maintained.

#### Research priorities

Immediate priorities are independent prospective validation of the Liu and Qiao models in multicenter cohorts outside their original development settings, with transparent reporting of calibration slope and intercept according to TRIPOD+AI. Minimum reporting requirements should include calibration slope and intercept with 95% confidence intervals, C-statistic with 95% confidence interval, decision curve analysis with net benefit, explicit threshold selection rationale, and public calculator or code availability. Short-term priorities include international collaborative networks for multilingual model validation and prospective impact studies using cluster-randomized, stepped-wedge, or pragmatic designs. Long-term priorities include incorporation of patient-reported outcomes and SONG-PD core domains, dynamic prediction models incorporating early reassessment markers, and model updating studies in diverse populations. We propose that future research follow the IDEAL framework for prediction model evaluation (24). Under this framework, all currently identified models appear to be in Stage 2a (development) or 2b (validation), with none reaching Stage 3 (prospective validation) or Stage 4 (clinical impact assessment).

While this review addresses a specialized nephrology topic, its implications extend to clinical prediction methodology more broadly. The findings illustrate a common pitfall in prognostic model development, namely the disconnect between statistical performance and clinical readiness, and our four-tier validation framework and calibration completeness criteria may be applicable beyond PD peritonitis.

## Conclusions

Based on the currently identified literature, and acknowledging potential language-related evidence gaps, currently no model has sufficient evidence for routine clinical implementation or screening; under strict supervision, local calibration, and a clear research purpose, some models may serve as research tools. Our most stringent standards require independent prospective external validation, complete calibration reporting (slope and intercept), and demonstrated impact on patient outcomes; all identified models fall short of these criteria. Two models (Liu et al., Qiao et al.) were rated low risk in the participants, predictors, and outcome domains but unclear overall owing to incomplete handling of missing data, lack of explicit overfitting assessment, and incomplete calibration reporting by our extended criterion; the remaining five models were rated high risk overall. Under a more inclusive validation definition, the proportion of models with any external validation would increase from 14% to 43%, but the fundamental conclusion remains unchanged: no model has undergone prospective validation or impact assessment, and complete calibration reporting is absent across all identified models. The most promising published models may be prioritized for further independent prospective validation, but the literature remains constrained by small study numbers, incomplete calibration reporting, limited external validation, potential language bias, and the absence of prospective impact assessment. We encourage the international nephrology community to establish collaborative networks for multilingual model validation and to prioritize patient-centered outcomes in future prediction model research.
