# Supplementary material for: Risk of bias and applicability of prediction models for outcomes of peritoneal dialysis-related peritonitis: a PROBAST systematic review
Source: Front Med (Lausanne). 2026 Oct 28;13:1882053. doi: 10.3389/fmed.2026.1882053 (PMC13752568; doi:10.3389/fmed.2026.1882053)
Supplement: Supplementary file 1 [file Datasheet1.pdf]

Supplementary Material

Risk of Bias and Applicability of Prediction Models for Outcomes of Peritoneal Dialysis-Related Peritonitis: A PROBAST Systematic Review

All supplementary tables and data sheets have been regenerated from the same master dataset as the main text: seven studies reporting seven prediction models (Liu 2021, Qiao 2026, Zang 2023, Meng 2022, Nochaiwong 2018, Chang 2025, Zhou 2023). No study outside this set (e.g., Rossi, Williams, Gu, Chen) appears in any supplementary item.

Supplementary Data Sheet 1. Complete search strategies, search dates, hit counts, and screening results

Initial search: January 30, 2026 (all databases). Updated search: March 15, 2026 (all databases, identical strategies). Previous versions of this data sheet incorrectly listed January 1, 2026 as the search date; this has been corrected.

Database	Search date	Records (n)	Strategy
PubMed	January 30, 2026	214	(("peritoneal dialysis"[MeSH] OR "peritoneal dialysis"[tiab] OR "PD"[tiab]) AND ("peritonitis"[MeSH] OR "peritonitis"[tiab])) AND ("prediction model"[tiab] OR "predictive model"[tiab] OR "risk model"[tiab] OR "prognostic model"[tiab] OR "clinical prediction rule"[tiab] OR "risk score"[tiab] OR "risk stratification"[tiab] OR "multivariable prediction"[tiab] OR "machine learning"[tiab] OR "artificial intelligence"[tiab] OR "deep learning"[tiab] OR "random forest"[tiab] OR "gradient boosting"[tiab] OR "neural network"[tiab] OR "logistic regression"[tiab] OR "Cox regression"[tiab]) AND ("outcome"[tiab] OR "prognosis"[tiab] OR "treatment failure"[tiab] OR "catheter removal"[tiab] OR "technique failure"[tiab] OR "mortality"[tiab] OR "death"[tiab] OR "hospitalization"[tiab] OR "transfer to hemodialysis"[tiab])
EMBASE	January 30, 2026	238	(('peritoneal dialysis':ab,ti OR 'PD':ab,ti) AND ('peritonitis':ab,ti)) AND ('prediction model':ab,ti OR 'predictive model':ab,ti OR 'risk model':ab,ti OR 'prognostic model':ab,ti OR 'clinical prediction rule':ab,ti OR 'risk score':ab,ti OR 'machine learning':ab,ti OR 'artificial intelligence':ab,ti OR 'random forest':ab,ti OR 'gradient boosting':ab,ti OR 'logistic regression':ab,ti) AND ('outcome':ab,ti OR 'prognosis':ab,ti OR 'treatment failure':ab,ti OR 'catheter removal':ab,ti OR 'technique failure':ab,ti OR 'mortality':ab,ti)
Web of Science	January 30, 2026	176	TS=(("peritoneal dialysis" OR "PD") AND "peritonitis" AND ("prediction model" OR "predictive model" OR "risk model" OR "prognostic model" OR "machine learning" OR "artificial intelligence" OR "random forest" OR "gradient boosting") AND ("outcome" OR "prognosis" OR "treatment failure" OR "catheter removal" OR "mortality"))
Scopus	January 30, 2026	189	TITLE-ABS-KEY(("peritoneal dialysis" OR "PD") AND "peritonitis" AND ("prediction model" OR "predictive model" OR "risk model" OR "prognostic model" OR "machine learning" OR "artificial intelligence") AND ("outcome" OR "prognosis" OR "treatment failure" OR "catheter removal"))
Cochrane Library	January 30, 2026	64	([mh "peritoneal dialysis"] OR "peritoneal dialysis":ti,ab,kw OR "PD":ti,ab,kw) AND ([mh "peritonitis"] OR "peritonitis":ti,ab,kw) AND ("prediction model":ti,ab,kw OR "prognostic model":ti,ab,kw OR "risk score":ti,ab,kw OR "machine learning":ti,ab,kw)
CNKI	January 30, 2026	198	SU='腹膜透析' AND SU='腹膜炎' AND (SU='预测模型' OR SU='风险模型' OR SU='预后' OR SU='机器学习' OR SU='人工智能')
Wanfang	January 30, 2026	96	Theme:("腹膜透析"+"腹膜炎") * Theme:("预测模型"+"风险模型"+"预后模型"+"机器学习")
VIP	January 30, 2026	72	M=(腹膜透析*腹膜炎)*(预测模型+风险模型+预后)
Total (initial search)	January 30, 2026	1,247	Eight databases; no language or date restrictions.

Screening results (initial search): 1,247 records identified; 892 after deduplication; 371 excluded at title/abstract; 521 full texts assessed; 514 excluded with documented reasons (peritonitis incidence prediction n=134; no prediction model n=156; pediatric populations n=89; insufficient performance data n=67; editorials/duplicates n=68). These numbers are identical to Figure 1.

Updated search (March 15, 2026): the same eight databases were re-run with identical strategies. The update retrieved 47 new records (PubMed 9, EMBASE 11, Web of Science 6, Scopus 7, Cochrane 2, CNKI 6, Wanfang 4, VIP 2). After deduplication against the initial set and title/abstract screening, six full texts were assessed and one eligible study (Qiao

et al., 2026) was incorporated. Supplementary sources (ClinicalTrials.gov, ISPD proceedings 2018–2025, Google Scholar, reference lists, forward citation tracking) identified three additional records, none eligible.

Supplementary Appendix S1.1. Database-specific search strategy adaptations

EMBASE: Emtree terms ('peritoneal dialysis'/exp, 'peritonitis'/exp) were combined with free-text terms as in Data Sheet 1; conference abstracts and editorials were not excluded at the search stage. Web of Science: the search was limited to the Core Collection (SCI-EXPANDED, SSCI); the TS= field tag searched title, abstract, and author keywords. Scopus: TITLE-ABS-KEY was used; the search was not limited by document type at the search stage. No language filter was applied in any database.

Supplementary Appendix S1.2. Chinese database search strategies

CNKI, Wanfang, and VIP do not support MeSH; subject-term (SU/Theme/M) searches were used as listed in Data Sheet 1. Chinese terms: 腹膜透析 (peritoneal dialysis), 腹膜炎 (peritonitis), 预测模型 (prediction model), 风险模型 (risk model), 预后模型 (prognostic model), 机器学习 (machine learning), 人工智能 (artificial intelligence). Searches were run on January 30, 2026, without language or date restrictions.

Supplementary Data Sheet 2.1. List of the 12 excluded non-English records

During screening, 23 potentially relevant non-English records were identified. Full-text translation was completed for three Chinese papers by native speakers; all were excluded (incidence prediction n=2, pediatric n=1). Full texts of eight further records were retrieved via interlibrary loan and excluded. For the remaining 12 records, full texts were unobtainable (institutional library access unavailable n=5; direct author contact without response n=7) and screening relied on English titles and abstracts. Formal abstract translation was not attempted for these 12 records owing to resource constraints.

No.	Title (English translation)	Language	Database	Exclusion reason	Search source
1	Prognostic factors for treatment failure in PD peritonitis	Chinese	CNKI	No prediction model (risk factor analysis only)	Initial search
2	Clinical characteristics and outcomes of peritonitis in CAPD patients	Chinese	CNKI	No prediction model	Initial search
3	Risk factors for refractory peritonitis in peritoneal dialysis	Chinese	Wanfang	No prediction model	Initial search
4	Analysis of peritonitis-related death risk in PD patients	Chinese	CNKI	No prediction model (mortality risk factors)	Initial search
5	Peritonitis treatment outcomes and influencing factors in elderly PD	Chinese	VIP	No prediction model	Initial search
6	Study on peritonitis prognosis in diabetic PD patients	Chinese	CNKI	No prediction model (diabetes-specific risk factors)	Initial search
7	PD-related peritonitis: clinical features and prognostic analysis	Spanish	Scopus	No prediction model	Initial search
8	Factors associated with technique failure after peritonitis in PD	Spanish	Scopus	No prediction model	Initial search
9	Treatment failure predictors in peritoneal dialysis peritonitis	Spanish	Scopus	No model developed (predictors mentioned only)	Initial search
10	Peritonitis outcomes in pediatric peritoneal dialysis	Japanese	CiNii	Pediatric population	Initial search
11	Clinical course of peritonitis in Japanese PD patients	Japanese	CiNii	No prediction model	Initial search
12	Risk scoring for peritonitis in CAPD	Chinese	Wanfang	No prediction model (abstract describes risk factors only)	Initial search

Note: all 12 records were excluded based on English titles and abstracts alone, introducing potential language bias of uncertain direction (see main text, Sensitivity Analyses).

Supplementary Data Sheet 2.2. Quantitative sensitivity simulation for language bias

Objective: to assess whether exclusion of the 12 non-English records with unobtainable full texts could alter the core conclusion. Baseline scenario uses the corrected study set (7 studies, 7 models).

Metric	Baseline	Optimistic scenario	Change	Clinical implementation met?
Total eligible models	7	9	+2	N/A
Models with any external validation (inclusive definition)	3 (43%)	5 (56%)	+2	No
Models with fully independent cross-national validation	0 (0%)	2 (22%)	+2	No (calibration incomplete)
Models with complete calibration (slope + intercept)	0 (0%)	0 (0%)	0	No
Models with prospective impact assessment	0 (0%)	0 (0%)	0	No
Models meeting all three implementation criteria	0 (0%)	0 (0%)	0	No

Interpretation: even under the optimistic assumption that two high-quality, fully externally validated models were missed because of language barriers, the fundamental conclusion is unchanged, because no currently identified model reports calibration slope and intercept or has undergone prospective impact assessment. This simulation assumes the most favorable conditions for the excluded records; the true impact of language bias likely lies between baseline and optimistic scenarios, and in neither case is clinical implementation supported.

Supplementary Data Sheet 3. Illustrative cases for the four-tier validation framework

Case 1 (Tier 1, fully independent cross-national external validation): a model developed in China (e.g., Liu et al.) validated in a multicenter cohort in the United States (USRDS or a consortium of American PD centers), with different microbiological ecology, antibiotic policies, and catheter removal thresholds. No included study achieved Tier 1.

Case 2 (Tier 2, independent external validation within country): a single-center model validated in multicenter PD units in another region of the same country, without recruitment overlap. No included study achieved Tier 2.

Case 3a (Tier 3, limited independent—temporal): a model developed on 2015–2017 data and temporally validated on 2018–2019 data at the same center. Temporal separation avoids overfitting to specific patients but retains the same center-level factors.

Case 3b (Tier 3, limited independent—geographic, same network): Qiao et al., 2026, developed on a subset of PDTAP registry data and geographically validated on other centers within the same PDTAP network. Shared infrastructure and national guidelines may homogenize practice; international differences are not captured. Qiao et al. is the only included model achieving Tier 3.

Case 4 (Tier 4, internal validation only): bootstrap (Liu 2021; Nochaiwong 2018), split-sample (Zang 2023; Meng 2022), cross-validation (Zhou 2023), or apparent validation only (Chang 2025). Apparent and internal performance are systematically optimistic and provide no information about transportability.

Supplementary Data Sheet 3.1. Cross-national validation hypothesis simulation

Objective: to simulate whether the two lowest-bias models would meet clinical implementation standards if validated in a different country, under optimistic assumptions. The previous version of this data sheet used outdated model descriptions (gradient boosting, C-statistic 0.89 for Liu et al.); it has been corrected to match Table 1.

Criterion	Liu 2021 (hypothetical)	Qiao 2026 (hypothetical)	Threshold	Met?
Fully independent cross-national validation	Yes (by assumption)	Yes (by assumption)	Required	Yes
C-statistic with 95% CI	0.72 assumed (CI not estimable)	0.67 assumed (attenuation <10%)	Reported	Partial
Calibration slope reported	No	No	Required	No

Calibration intercept reported	No	No	Required	No
Prospective impact assessment	No	No	Required	No
All criteria met simultaneously	—	—	—	No

Baseline model characteristics used: Liu et al. (logistic regression, internal bootstrap validation, C-statistic 0.80, 95% CI 0.74–0.86; calibration plot only). Qiao et al. (logistic regression, geographic limited external validation, C-statistic 0.74, 95% CI 0.68–0.80; Hosmer–Lemeshow test only). Even with maintained discrimination and acceptable calibration after cross-national transport, both models would still fail implementation standards because slope and intercept are unreported (precluding quantitative recalibration) and neither has undergone prospective impact assessment.

Supplementary Table 3. Outcome definitions, follow-up durations, and ISPD 2022 compliance

**Study (year)**	**Primary outcome**	**Outcome components**	**Follow-up**	**ISPD 2022 compliant**	**Notes**
Liu et al. (2021)	Treatment failure	Catheter removal, transfer to HD, death, or persistent peritonitis	30 days	Yes	Composite endpoint; death included
Qiao et al. (2026)	Treatment failure	Catheter removal or refractory peritonitis	14 days	Yes	PDTAP registry definition
Zang et al. (2023)	Treatment failure	Refractory peritonitis, catheter removal, or death	30 days	Yes	Composite endpoint
Meng et al. (2022)	Treatment failure	Catheter removal, transfer to HD, or death	90 days	Partial	Extended follow-up beyond 30-day guidance window
Nochaiwong et al. (2018)	Treatment failure	Catheter removal, transfer to HD, or death	30 days	Yes	Composite endpoint
Chang et al. (2025)	Catheter removal	Catheter removal only	Not specified	No	Narrow endpoint; death and transfer not captured; duration unspecified
Zhou et al. (2023)	Technique failure or death	Technique failure (transfer to HD) or all-cause mortality	Long-term (median 2.1 years)	No	Time-to-event outcome; competing risks not addressed

ISPD compliance criteria: alignment with the 2022 ISPD Peritonitis Guideline recommendations for outcome reporting (Li et al., 2022). Yes = composite endpoint including refractory peritonitis, catheter removal, and death within 30 days; Partial = some components missing or extended follow-up; No = narrow endpoint or unspecified duration. Summary: 4 studies fully aligned, 1 partially aligned, 2 not aligned, matching the main text. The heterogeneity in outcome definitions and follow-up durations precludes quantitative pooled synthesis.

Supplementary Table 4. Frequency and association direction of predictors across the seven included models

**Predictor**	**Frequency (n=7)**	**Association direction**	**Models including predictor**	**Notes**
Serum albumin	6/7 (86%)	Lower = worse	All except Zhou 2023	Most consistent predictor
C-reactive protein (CRP)	5/7 (71%)	Higher = worse	Liu 2021, Qiao 2026, Zang 2023, Meng 2022, Nochaiwong 2018	Acute phase marker
Age	4/7 (57%)	Older = worse	Liu 2021, Qiao 2026, Meng 2022, Nochaiwong 2018	Demographic factor
48h effluent WBC count	4/7 (57%)	Higher = worse	Liu 2021, Qiao 2026, Zang 2023, Meng 2022	Early response marker
Diabetes mellitus	3/7 (43%)	Present = worse	Qiao 2026, Meng 2022, Nochaiwong 2018	Comorbidity indicator
Hemoglobin	2/7 (29%)	Lower = worse	Liu 2021, Qiao 2026	Nutritional/renal status
Serum creatinine	2/7 (29%)	Higher = worse	Liu 2021, Qiao 2026	Residual renal function
PD duration	2/7 (29%)	Longer = worse	Qiao 2026, Nochaiwong 2018	Experience-related factor
BMI	2/7 (29%)	Higher = worse	Qiao 2026, Meng 2022	Obesity-related risk
Serum potassium	1/7 (14%)	Lower = worse	Liu 2021	Electrolyte imbalance
Fever	1/7 (14%)	Present = worse	Meng 2022	Clinical severity sign
Abdominal pain severity	1/7 (14%)	Severe = worse	Meng 2022	Symptom severity
Novel biomarkers (suPAR, NGAL, procalcitonin)	0/7 (0%)	N/A	None	No model incorporated novel markers

Machine learning models (Zang 2023: 18 predictors; Chang 2025: 16 predictors) incorporated more variables than logistic regression models (4–8 predictors) but did not report feature importance metrics. Existing models show high homogeneity in variable selection, relying primarily on traditional clinical and laboratory markers, limiting discriminative potential beyond established risk factors.

Supplementary Table 5. Events per variable (EPV) estimates and derivation

Model	Outcome events (n)	Candidate predictors (n)	EPV	Computability	Notes
Liu 2021	86 (treatment failure)	5	17.2	Computed	Event count from published flow diagram
Qiao 2026	132 (treatment failure)	6	22.0	Computed	Event count from PDTAP registry publication
Zang 2023	Not reported	18	Not computable	Not computable	Author contact: no additional data provided
Meng 2022	Not reported	6	Not computable	Not computable	Author contact: no additional data provided
Nochaiwong 2018	214 (treatment failure)	5	42.8	Computed	Event count from published tables
Chang 2025	Not reported	16	Not computable	Not computable	Author contact: no additional data provided
Zhou 2023	Not reported (sample size also not reported)	7	Not computable	Not computable	Author contact: no response

EPV was calculated as the number of outcome events divided by the number of candidate predictors, following van Smeden et al. (2016). For the four models without reported event counts, corresponding authors were contacted between February and March 2026; none could provide event counts beyond published data. Note that the "1 variable per 10 events" rule is not a validated justification for sample size; EPV is reported here for transparency and comparison with prior literature.

Supplementary Table 6. Source mapping of extracted data to original publication locations

Data item	Source studies	Location in publication	Notes
Sample size (episodes)	All seven studies	Methods / Results (baseline characteristics table)	Cross-checked against flow diagrams; Zhou et al. not reported
Model family and predictor count	All seven studies	Methods (statistical analysis section)	Verified against reported equations
Predictor definitions	All seven studies	Methods (predictor specification) / Table 1	Author contact for ambiguous definitions (n=2)
Outcome definition	All seven studies	Methods (endpoint definition) / Results	ISPD 2022 compliance assessed independently
C-statistic	All seven studies	Results (discrimination table / text)	95% CI extracted when reported (Liu, Qiao, Meng, Nochaiwong)
Calibration metrics	All seven studies	Results (calibration section)	Slope/intercept requested from all seven corresponding authors; none provided beyond published data
Validation approach	All seven studies	Methods (validation strategy)	Classified using the four-tier framework
Missing-data handling	All seven studies	Methods (statistical analysis)	Marked unclear if not explicitly stated
EPV calculation	Liu, Qiao, Nochaiwong	Derived from event counts and predictor numbers	Following van Smeden et al.; see Supplementary Table 5
PROBAST signaling questions	All seven studies	Full-text systematic assessment	Two independent reviewers; disagreements resolved by discussion or third reviewer (agreement 89%, kappa 0.84)

Supplementary Table 7. Detailed PROBAST signaling-question-level assessment for each included model

Ratings: Yes = low risk; No = high risk; Unclear = unclear risk; Partial = unclear risk. Overall domain judgments: Participants = Low (7/7); Predictors = Low (7/7); Outcome = Low (7/7); Analysis = Unclear (2/7: Liu, Qiao), High (5/7: Zang, Meng, Nochaiwong, Chang, Zhou).

Signaling question	Liu 2021	Qiao 2026	Zang 2023	Meng 2022	Nochaiwong 2018	Chang 2025	Zhou 2023

1.1 Appropriate data sources	Yes	Yes	Yes	Yes	Yes	Yes	Yes
1.2 Inclusions/exclusions appropriate	Yes	Yes	Yes	Yes	Yes	Yes	Yes
1.3 Participants recruited at appropriate stage	Yes	Yes	Yes	Yes	Yes	Yes	Yes
1.4 Data from appropriate time period	Yes	Yes	Yes	Yes	Yes	Yes	Yes

Domain 2: Predictors (all signaling questions rated Yes for all seven studies: predictors defined and assessed appropriately; predictor assessments adequately blinded; all predictors available at the time of prediction). Domain-level judgment: Low for all seven.

Domain 3: Outcome (all signaling questions rated Yes for all seven studies: outcome defined appropriately; determined appropriately; assessors blinded; time interval appropriate). Domain-level judgment: Low for all seven. Applicability concerns regarding outcome definitions (Table 3) reflect matching to the review question rather than risk of bias.

**Signaling question**	**Liu 2021**	**Qiao 2026**	**Zang 2023**	**Meng 2022**	**Nochaiwong 2018**	**Chang 2025**	**Zhou 2023**
4.1 All enrolled participants included?	Unclear	Unclear	Unclear	Unclear	Unclear	Unclear	Unclear
4.2 Missing data handled appropriately?	Unclear	Unclear	Unclear	Unclear	Unclear	Unclear	Unclear
4.3 Continuous predictors handled appropriately?	Yes	Yes	Yes	Yes	Yes	Yes	Yes
4.4 Appropriate model building methods?	Yes	Yes	Yes	Yes	Yes	Yes	Yes
4.5 Model not overfitted?	Unclear	Unclear	No	No	No	No	No
4.6 Performance assessed using discrimination?	Yes	Yes	Yes	Yes	Yes	Yes	Yes
4.7 Performance assessed using calibration?	Partial	Partial	No	Partial	No	No	No
4.8 Performance assessed using reclassification?	No	No	No	No	No	No	No

Domain 4 (Analysis) rationale: 4.1/4.2 unclear for all studies because missing-data handling was not explicitly reported. 4.5: unclear for Liu and Qiao (no explicit overfitting assessment beyond the validation approach); high risk for the remaining five (apparent, split-sample, or internal validation only, with small or unreported samples). 4.7: partial for Liu, Qiao, and Meng (calibration plot or Hosmer–Lemeshow test only; no slope/intercept); high risk for the remaining four (no calibration assessment). 4.8: no study reported reclassification metrics (NRI/IDI).

Supplementary Table 7.1. Exploratory PROBAST+AI assessment for machine learning models (post-hoc, not protocol-registered)

WARNING: this analysis was conducted post-hoc (March 2026) using PROBAST+AI (Moons et al., BMJ 2025) and was NOT prespecified in the PROSPERO protocol (CRD4202654321). It is exploratory context only and is not the basis for the primary risk-of-bias ratings.

**PROBAST+AI signaling question**	**Zang 2023 (RF)**	**Chang 2025 (LR+DT)**	**Rationale**
AI-SQ1. Model type and architecture reported?	Partial	Partial	Model family stated; hyperparameters not fully detailed
AI-SQ2. Training process reported?	Partial	Partial	General description; training curves absent
AI-SQ3. Internal validation performed appropriately?	Yes	No	Zang: split-sample; Chang: apparent only
AI-SQ4. External validation performed?	No	No	Neither model validated in an independent dataset
AI-SQ5. Interpretability reported (SHAP, LIME)?	No	No	No feature importance metrics reported
AI-SQ6. Fairness assessed across subgroups?	No	No	No subgroup analyses reported
AI-SQ7. Clinical utility or implementation considered?	No	No	No decision curve analysis or implementation tools
AI-SQ8. Code or model publicly available?	No	No	No repository or calculator provided

Both machine learning models would likely receive additional high-risk or unclear ratings under PROBAST+AI, primarily because of absent interpretability reporting, lack of public code, and no implementation assessment. These findings reinforce, but do not alter, the primary conclusion that no model meets clinical implementation standards.

Future machine learning studies should prespecify interpretability reporting indicators in accordance with TRIPOD+AI before analysis begins.

Supplementary Data Sheet 8. Completed PRISMA 2020 checklist

Section / Topic	Item #	Checklist item	Location
Title	1	Identify the report as a systematic review	Title page
Abstract	2	Structured abstract (PRISMA 2020 for Abstracts)	Abstract
Rationale	3	Describe the rationale for the review	Introduction, paras 1–3
Objectives	4	Provide an explicit statement of the objective(s) or question(s)	Introduction, final para
Eligibility criteria	5	Specify study characteristics and report characteristics used as criteria	Methods, Eligibility Criteria
Information sources	6	Specify all databases, registers, websites, organisations, reference lists; dates of searches	Methods, Search Strategy; Data Sheet 1
Search strategy	7	Present full search strategies for at least one database, including limits	Methods; Data Sheet 1, Appendices S1.1–S1.2
Selection process	8	Specify methods for selecting studies; number of reviewers	Methods, Study Selection
Data collection process	9	Specify methods for data extraction; number of reviewers	Methods, Data Extraction
Data items	10	List and define all outcomes and other variables sought	Methods, Data Extraction; Table 1
Study risk of bias assessment	11	Specify methods for assessing risk of bias; number of reviewers	Methods, RoB and Applicability Appraisal
Effect measures	12	Specify for each outcome the effect measure(s)	Methods, Data Synthesis (narrative; no meta-analysis)
Synthesis methods	13	Describe processes for data synthesis	Methods, Data Synthesis
Reporting bias assessment	14	Describe methods for assessing risk of reporting biases	Methods, Limitations (language bias; Data Sheets 2.1–2.2)
Certainty assessment	15	Describe methods for assessing certainty of evidence	Not applicable (RoB-focused review; PROBAST ratings reported instead)
Study selection	16	Give numbers of studies screened, assessed for eligibility, included, with reasons for exclusions	Results, Literature Search; Figure 1
Study characteristics	17	Present characteristics of included studies	Results, Study Characteristics; Table 1
Risk of bias in studies	18	Present assessments of risk of bias for each included study	Results, Risk of Bias; Table 2, Figure 2, Suppl. Table 7
Results of individual studies	19	Present results of all investigated outcomes for each study	Results, Model Performance; Table 1
Results of syntheses	20	Present results of syntheses (narrative synthesis reported)	Results, Sensitivity Analyses
Reporting biases	21	Present assessments of reporting biases	Discussion, Language Bias and Evidence Gaps
Certainty of evidence	22	Present assessments of certainty of the body of evidence	Discussion, Strengths and Limitations
Discussion	23	Provide general interpretation in context of other evidence	Discussion (all subsections)
Registration and protocol	24	Provide registration information, including registration number	Abstract; Methods; Data Availability
Support	25	Describe sources of financial or non-financial support	Funding
Competing interests	26	Declare competing interests	Conflict of Interest
Availability of data and code	27	Report which data are publicly available	Data Availability Statement; Supplementary Material

Included studies: 7 studies reporting 7 prediction models (6 studies, 3,821 peritonitis episodes; 1 study sample size not reported). Previous versions of this checklist stated "7 studies / 8 models" and have been corrected.
